# Photosystem I Photopolymerizes Pyrrole into Spherical Nanocomposites

**DOI:** 10.1021/acs.biomac.5c00263

**Published:** 2025-04-21

**Authors:** William R. Lowery, Allison C. Portaro, G. Kane Jennings, David E. Cliffel

**Affiliations:** † Department of Chemistry, Vanderbilt University, Nashville, Tennessee 37235-1822, United States; ‡ Department of Chemistry, University of Louisville, Louisville, Kentucky 40292, United States; § Department of Chemical and Biomolecular Engineering, Vanderbilt University, Nashville, Tennessee 37235-1604, United States; ∥ Department of Chemistry, Vanderbilt University, Nashville, Tennessee 37235-1822, United States

## Abstract

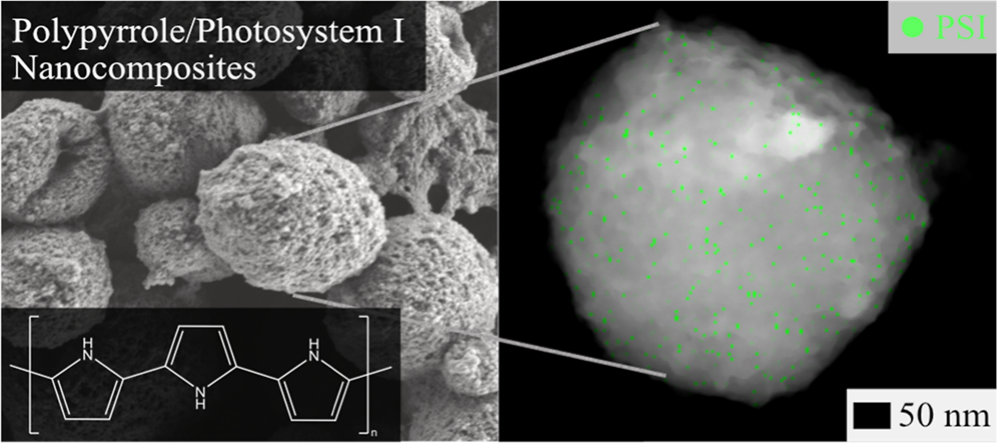

Conductive polymers have been shown to be an effective scaffold for proteins when designing bioelectrochemical systems, particularly for the Photosystem I protein. Utilization of synthetic polymer chemistry has allowed a great deal of tunability within the protein/polymer interface to improve electron transfer from the proteins, ultimately progressing toward direct electron transfer from the active sites. Seeking to address this issue, a new heterogeneous approach is presented to synthesize Photosystem I/polypyrrole (PSI/PPy) composites. The oxidative potential of PSI’s P_700_ reaction site was leveraged to polymerize pyrrole into a molecular wire, providing a more efficient means of electron transfer to the protein. Over the course of several hours of photopolymerization of Py in a PSI film, PPy not only wired PSI but began incasing the protein within conductive polymer nanoparticles. These resulting composite nanoparticles were extensively characterized by electron microscopy and electrochemical techniques to showcase their synergistic properties.

## Introduction

Photosystem I (PSI), a transmembrane protein found in most photosynthetic organisms, has been studied extensively over the years due to its ubiquity in nature, ex vivo stability, and unique photoelectrochemical properties.^1−5^ Of particular note are PSI’s near perfect internal quantum efficiency and potential difference of over 1 V, making it an excellent electron promoter.^4,5^ The driving force of PSI has been leveraged for the production of hydrogen^6,7^ and the reduction of carbon dioxide^8,9^ in a photosynthetically inspired fashion. These processes are accomplished by the redox active sites, P_700_ and F_b_. The P_700_ site exhibits an oxidative potential of +300 mV vs Ag/AgCl whereas the F_b_ site holds nature’s strongest reducing potential of −700 mV vs Ag/AgCl. Both potentials hold great opportunity to drive electrochemical processes. Some of these opportunities are limited though, due to the insulative nature of a bulky protein, and would benefit greatly by interfacing other materials with the electron donor/acceptor site.

Combining proteins such as PSI with conductive polymers has led to an exciting class of biohybrid materials that have been utilized in a wide variety of applications, from solar active layers^10,11^ to catalysis.^12^ Synthesis schemes for these composites have utilized approaches such as entrapment during electrochemical deposition,^10^ encapsulation within biopolymers,^13^ and chemical deposition through the vapor phase.^14^ These composites are designed to foster efficient electron transfer from the electroactive sites of the protein to the conductive framework. Ideally, Photosystem I would be able to perform direct electron transfer to the interfacing conductive polymer, removing the need for electron mediators.^15^

Providing a means to directly wire the photosystem’s reaction sites not only accomplishes direct electron transfer, but it would also provide a tether for furthering orientation schemes. Previous work has focused on selectively modifying PSI to introduce chemical functionality allowing for controlled orientation relative to an underlying substrate.^16,17^ Through introduction of an electrochemically active chain from one end of PSI, avenues for both orientation and direct electron transfer is achievable.

A stepping stone toward direct electronic transfer was made through utilization of PSI’s oxidative potential to grow a conductive polymer chain from the P_700_ site using a homogeneous, photooxidative reaction in aqueous solution.^18^ Growth of the conductive polymer, polypyrrole, yielded composites that exhibited improved stability while maintaining PSI’s photoactivity. Composite protein polymer conjugates such as these have been used widely to accomplish a variety of tasks and have spurred many studies on their synthesis.^19−21^ In one case, polymers are conjugated to therapeutic proteins to reduce the dosage frequency in patients.^22^ In another report, polymers are shown to not only protect the activity of enzymes but also improve it.^23^ In almost every field that proteins are used lie opportunity to apply enhanced conjugates.

**Figure 1:**
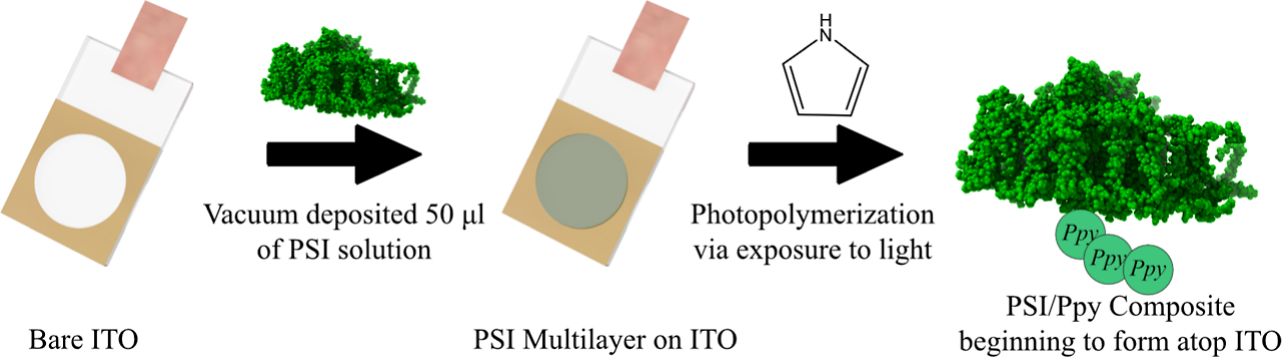
Mechanistic overview of the experimental aspects of photopolymerization. A multilayer of Photosystem I was deposited atop a conductive ITO substrate. The substrate was then submerged in a solution of pyrrole monomer and exposed to light for varying time points to allow for PPy growth to form PSI/PPy composites.

In this spirit, we advance the previous approach to synthesize a PSI polymer conjugate to a heterogeneous strategy by utilizing a multilayered film of PSI as opposed to solution-based PSI to obtain composite thin films and nanoparticles that exhibit improved photoactivity when compared to the homogeneously synthesized composites.^18^ Other synthetic routes for conductive polymer nanoparticles have been reported in recent years, which have been motivated by a desire to generate biocompatible conductive polymer nanocomposites.^24−27^ Herein, we present a photooxidative methodology (as shown below by Figure 1 and the accompanying reactions) to synthesize polypyrrole/PSI nanoparticles that synergistically improves upon PSI’s photoactivity.
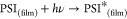

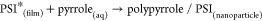


## Experimental Section

### Photosystem I Extraction

Store bought spinach was utilized for the extraction of Photosystem I (PSI) with a procedure previously reported by Baba et al.^3,28^ The PSI containing thylakoids were isolated via a postblended centrifugation step. Following centrifugation, the slurry was loaded onto a hydroxyapatite ion exchange column to isolate PSI from other cellular components.^29^ Photosystem I was then eluted from the column through addition of a higher ionic strength buffer along with surfactant Triton X-100 (TX-100). This procedure yielded protein at concentration of ∼2 mg/mL as quantified by a spectroscopic method and was then stored in −80 °C until used.^3^ Finally, the protein was dialyzed in a 1:1000 ratio against water in a 10 kDa MWCO membrane to remove excess surfactant and promote protein interaction with ITO substrate.

### Photopolymerization Design

Initially, ITO substrates purchased from Ossila Ltd. (London, UK) were cleaned with isopropanol and then dried with compressed air. PSI multilayers were subsequently deposited via vacuum-assisted dropcasting of 25–75 μL of dialyzed solution. The multilayer substrates were then placed in glass Petri dishes with accompanying glass covers and submerged within pyrrole monomer solution (0.5 M pyrrole and 1.0 M NaClO_4_ as the counteranion/electrolyte) for illumination with a Schott KL 1500 HAL for a varied amount of time. Red light illumination was performed by adding a Schott red lens filter to focus the illumination on wavelengths that more selectively promoted excitation within PSI. Finally, experiments on deactivated PSI were performed after the PSI solution was boiled for 20 min and exposed to UV light for 6 h to yield a deactivated complex. Chemical signatures of resulting composites were probed via FTIR and are included in Figure S1.

### Chemical Polymerization of Polypyrrole

Pure polypyrrole was synthesized through use of chemical oxidant iron(III) chloride for spectroscopic comparison with protein polymer composites.^30^ Pyrrole (1.5 g) was initially dissolved in a round-bottom flask with 150 mL of water. Iron(III) chloride (15 g) was added to the reaction flask after dissolution in 30 mL of water. The flask was capped and allowed to stir for 24 h at ambient conditions. The reaction produced black precipitates which were then centrifuged for removal of unreacted reactants. The crude mixture was separated into conical tubes and centrifuged to pellet the desired polymer product. Supernatant containing oligomers, pyrrole, and iron were discarded over the course of ten cycles. After discarding the supernatant, water was readded to the flask and sonicated to redisperse the pellet between centrifugation cycles.

### Electrochemical Characterization

Electrochemical measurements were obtained with a CHI 660A workstation (CH Instruments, Austin, TX). The ITO substrates were utilized as the working electrode, whereas a platinum mesh was used for the counter electrode, and Ag/AgCl was used as the reference electrode. Photochronoamperometry measurements were taken with a 2,6-dichlorophenolindophenol sodium salt/sodium ascorbate (DCPIP/NaAsc) mediator pair (0.1 mM DCPIP, 0.5 mM NaAsc) at the open circuit potential (OCP) upon illumination with a Schott KL 2500 LCD light source.

### Electron Microscopy Imaging

Scanning electron microscopy (SEM) was performed on a Zeiss Merlin system. A beam of 2 kV and 100 pA was utilized for imaging at a distance ∼5 mm following Au sputter coating with a Cressington 108 Sputter Coater. Scanning/transmission electron microscopy (S/TEM) was performed using an FEI Tecnai G2 Osiris system. TEM samples were prepared by sonicating PSI/Ppy films to disperse the material into 5 mL of DI water. The solution was then centrifuged down to concentrate the material into 100 μL. Finally, Lacey Formvar carbon-coated copper grids (obtained from Ted Pella) were briefly dipped for 3 rounds of 3 s into the solution to obtain a sample for imaging.

### Polypyrrole Control Nanoparticles

Polypyrrole nanoparticles were synthesized for use in comparison of PSI grown nanoparticles. The procedure was based upon a previously reported synthetic route.^31^ Initially, 14 mmol of anhydrous FeCl_3_ and 3 mmol of polyvinylpyrrolidone (PVP), *M*_w_ = 10,000, were stirred rapidly in a 1:4 ratio of 40 mL of ethanol to water. After 1 h, 6 mmol of pyrrole dissolved in 10 mL of DI water was added dropwise to the stirring solution. The stirring was then slowed to 400 rpm and continued for approximately 4 h. Finally, the dark black solution was centrifuged and washed with ethanol until the supernatant became clear. TEM samples were prepared by diluting 100 μL of the crude reaction mixture to 2 mL until a faint black color remained and dipping lacy carbon-coated copper grids into the diluted sample.

## Results and Discussion

**Figure 2:**
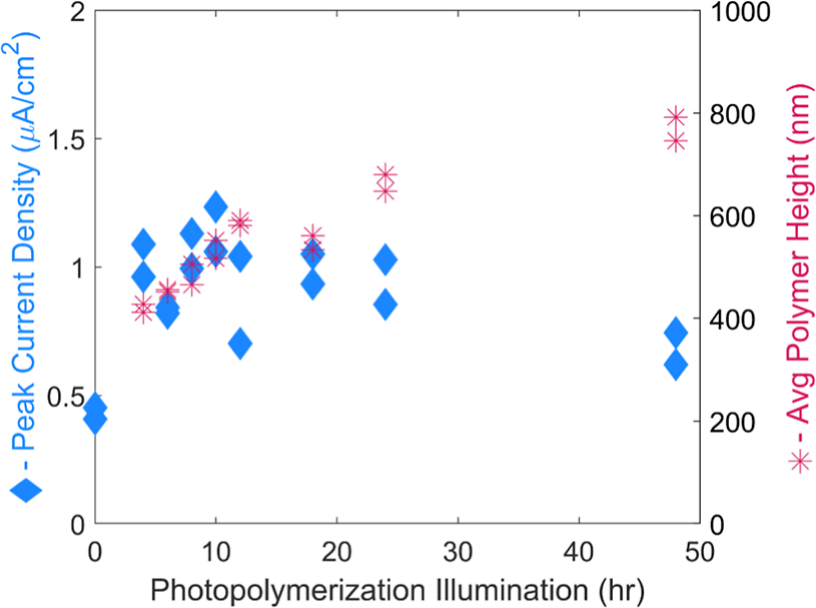
Photochronoamperometry (blue, left axis) for PSI/PPy composite films grown from drop casted 50 μL of 2 mg/mL PSI solution on ITO with accompanying profilometry (red, right axis) of polymer growth. More than doubled photocurrent is seen upon initial modification with PPy. Further improvement is minimal despite a growing film of PPy over the course of 48 h.

After several composite films of varying thicknesses were made through the photopolymerization of PPy by PSI multilayers, the effect of polymerization time was studied with photochronoamperometry (PCA). PCA examines the current output from the applied photopotential of PSI upon illumination with a solar simulator. Here, the PCA can be used as a metric to compare how the PPy modifications alter the electron transfer dynamics to and from the protein’s reaction centers. The photocurrent increases sharply for photopolymerization times from 0 to 4 h but exhibits little to no change for longer times, as shown in Figure 2. This jump represents the initial modification of Ppy wiring to the P_700_ reaction site, thereby increasing the electron net for the mediator to donate through. Once this initial chain is established, there is little improvement in photocurrent, despite further growth of the polymer.

**Figure 3:**
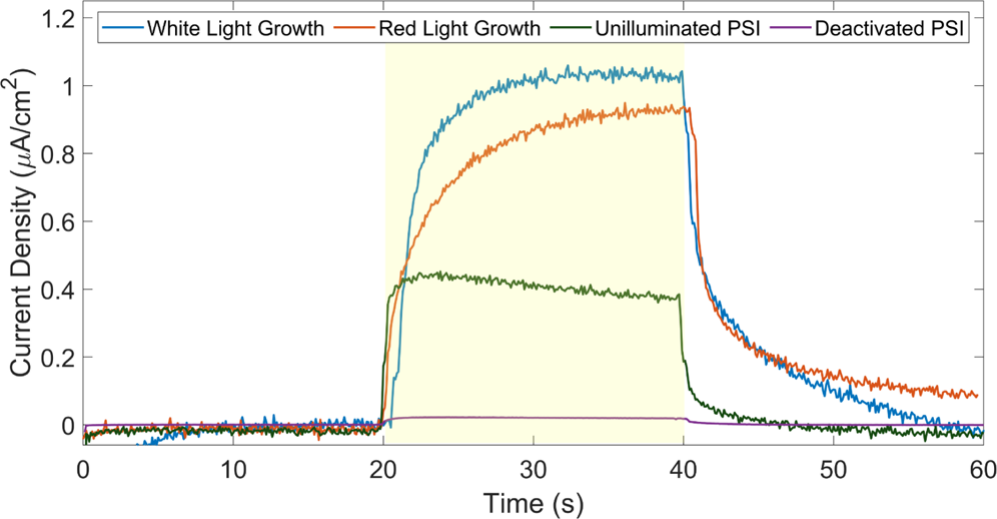
Photochronoamperometry for composite films prepared by exposure of Py to PSI for 24 h in white or red light, a deactivated PSI film exposed to the same conditions, and an unilluminated, Py-exposed PSI control. Measurements were taken at the OCP of each film with a 0.1 mM DCPIP, 0.5 mM NaAsc mediator pair. Illumination was carried out from 20 to 40 s.

An additional series of PCA experiments were also performed on films illuminated for 24 h to further the understanding of photopolymerization. Here, four different scenarios were explored that could potentially alter photopolymerization. Across these four scenarios a red light illumination, a UV-deactivated PSI, and an unilluminated PSI that were all exposed to Py monomer were compared to the standard white light illuminated film of a PSI multilayer that was not illuminated during Py exposure to compare how the resulting photoactivity was altered. A red filter illuminated the film under red light, focusing the photons on the excitation wavelength of P_700_. The resulting PCA in Figure 3 shows little change when compared to the normal white light photopolymerization. Also included in the graph is a measurement of a deactivated film of PSI. A solution of PSI was deactivated as described in the experimental section prior to an attempted photopolymerization. The negligible current when compared to the PSI control supports that the protein was indeed deactivated and unable to polymerize Py. These experiments support the fact that PPy is photopolymerized by the function of PSI and not just the presence of a protein.

**Figure 4:**
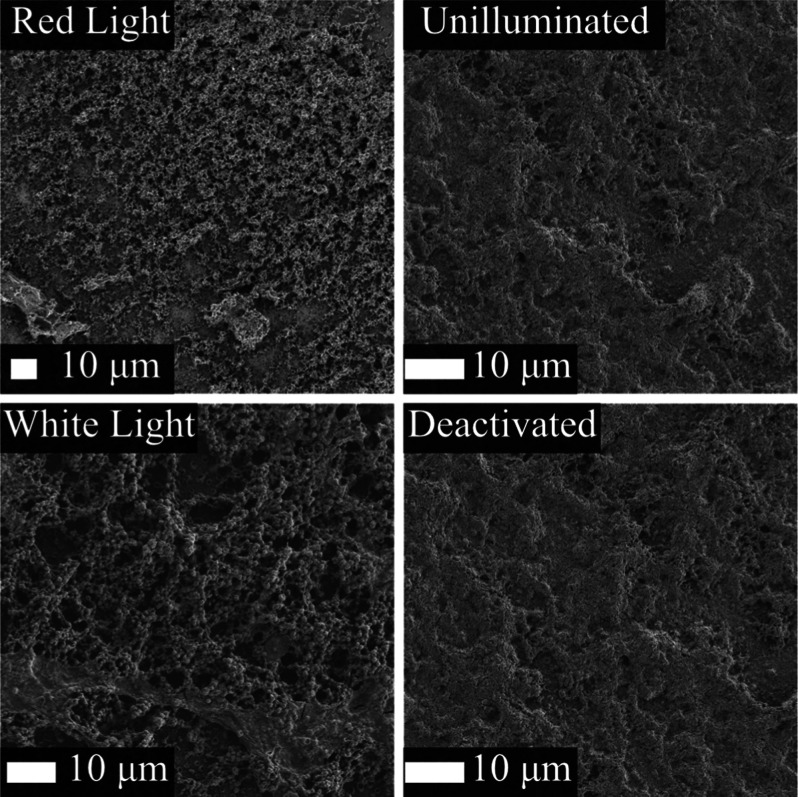
SEM micrographs of red and white light photopolymerization of PPy along with PSI control and deactivated PSI films where image labels represent conditions during the exposure with pyrrole for photopolymerization.

SEM characterization was utilized to further our understanding of the polymerization parameters beyond the conclusions that PCAs could provide. The SEM micrographs in Figure 4 of PSI–PPy composites prepared from white and red-light illumination did not exhibit any significant differences. Both films exhibited a globular microstructure, suggesting that the red-light illumination which focuses the irradiation on the absorbance band of P_700_ did not alter the growth mechanism. Imaging of a deactivated PSI film exposed to white illumination in the presence of Py monomer was also compared to an unilluminated PSI film control. In both micrographs, a rough surface is observed. This similarity suggests that PPy growth is controlled by the electrochemistry provided by the reaction centers and not simply the illumination of Py and nucleation on the protein surface.

**Figure 5:**
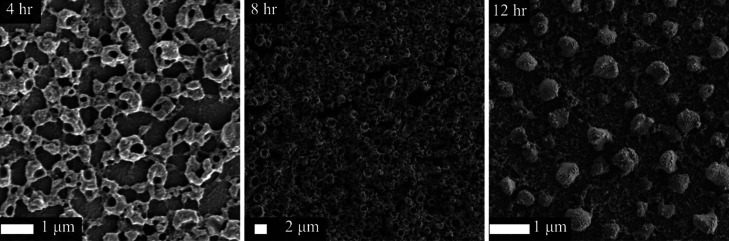
SEM images of PPy/PSI films with 4, 8, and 12 h of white illumination of PSI multilayer exposed to Py at a concentration of 0.5 M.

The morphology of the growth over the span of 12 h was tracked via SEM as shown in Figure 5. Initially, a column or barnacle-like structure occurs as the PPy began to grow through the PSI multilayer. This morphology is rather similar to that which we reported recently by electropolymerizing Py through the voids of PSI multilayers.^32^ Over the next few hours of growth, the structure begins to pack more densely until a honeycomb-like organization is established. As the growth continues to 10 and 12 h, the morphology shifts to enclose the columns into spheres. A more expansive selection of SEM images is given in Figure S2 along with a histogram of sizes in Figure S3.

**Figure 6:**
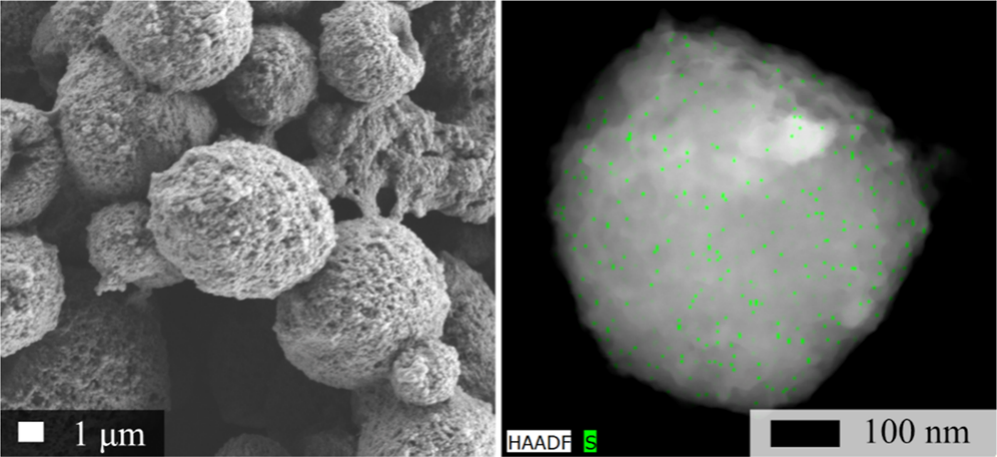
SEM and STEM images highlighting the spherical morphology of photopolymerization times >12 h. The panel on the left shows an SEM image of a film before it was removed for STEM imaging. The STEM EDX image on the right introduces a green highlight to the presence of sulfur suggesting PSI.

To further investigate the spherical morphologies, scanning/transmission electron microscopy (S/TEM) coupled with energy dispersive X-ray spectroscopy (EDX) was leveraged to determine the composition of the spheres in a more isolated environment (Figure 6). The STEM EDX panel highlights the presence of PSI within the individual particle through tracking of sulfur, a methodology that has previously been used to identify PSI.^14^ The uniform distribution of sulfur, which is a principal component to PSI’s structure, suggests that PSI is evenly distributed throughout the composite. The full EDX spectrum is given in Figure S4 along with additional images of other morphological remnants, such as columns and numerous other spheres in Figures S5 and S6. This composite PSI/PPy structure is envisioned to be a somewhat spherical composite of polypyrrole which grew around the PSI proteins. It is hypothesized that while pyrrole was photopolymerized by PSI, neighboring composites began to coalesce into larger units, ultimately forming the observed particles.

Control PPy nanoparticles (synthesized through more traditional chemical means) were comparatively characterized to validate that a sulfur signal could not be arising from the polypyrrole. In Figure S6, TEM is presented of the undiluted reaction mixture indicating monodispersity of the sample. The PPy/PVP nanoparticles were then diluted to allow for single particle imaging with STEM. This single particle is presented in Figure S7 along with the corresponding EDX spectra in Figure S8. The spectrum notably does not exhibit a sulfur signature. The use of EDX sulfur tracking as a means for PSI is supported with this comparative characterization of PPy/PVP.

## Conclusion

Photosystem I was shown to be an excellent photocatalyst for polymerization of pyrrole. Through this direct oxidative polymerization, the P_700_ reaction site was able to be wired with polypyrrole generating a much larger surface area with which to shuttle electrons into the electron transport mechanism. Furthermore, by leveraging multilayered films of PSI for the growth platform of PPy, an interesting growth mechanism was discovered leading to the discovery of a PSI/polymer nanoparticle. These PSI/polymer composites will undoubtedly lead to great interest in the field as a new means with which to interface PSI as opposed to the classic multilayered approach that has been extensively used over the years.

## Supporting Information Available

FTIR, SEM of PSI/PPy Films, SEM particle sizes histogram, EDX spectra, TEM and STEM of other morphologies, and TEM/EDX of PPy control nanoparticles (PDF)

## Abbreviations

PSI: photosystem I
PSII: photosystem II
Triton X-100: TX-100
FTIR–ATR: Fourier transform infrared spectroscopy–attenuated total reflectance
SEM: scanning electron microscopy
TEM: transmission electron microscopy
STEM: scanning transmission electron microscopy
PCA: photochronoamperometry
OCP: open circuit potential
ITO: indium tin oxide
EDS/EDX: Energy dispersive X-ray spectroscopy
