# Supplementary material for: Photosystem I Photopolymerizes Pyrrole into Spherical Nanocomposites
Source: Biomacromolecules. 2025 Apr 21;26(5):3180–5. doi: 10.1021/acs.biomac.5c00263 (PMC12076494; doi:10.1021/acs.biomac.5c00263)
Supplement: Supplementary file 1 — bm5c00263_si_001.pdf [file bm5c00263_si_001.pdf]

Supporting Information

Photosystem I Photopolymerizes Pyrrole into Spherical Nanocomposites

William R. Lowery[1], Allison C. Portaro[2], G. Kane Jennings[3], and David E. Cliffel[1]

[1]Department of Chemistry, Vanderbilt University, Tennessee 37235-1822, United States;

[2] Department of Chemistry, University of Louisville, Kentucky, 40292, United States

[3]Department of Chemical and Biomolecular Engineering, Vanderbilt University, Tennessee 37235-1604, United States.

Infrared spectroscopy was performed on samples with a Thermo Fisher Nicolet is20 spectrometer with an ATR accessory. Films were scraped off of ITO glass substrate with the edge of a razor blade to be used to coat the ATR diamond crystal for measurement. Spectra were obtained with 256 scans at a resolution of 4 cm^{-1}.

The composition of Photosystem I/polypyrrole (PSI/PPy) films showed signatures of both PSI and pyrrole absorption bands as characterized by FTIR-ATR. Spectra of pure PSI, chemically polymerized PPy, and proposed PSI/PPy are shown in **SI1**. The PSI spectrum shows the presence of both Amide I (1630 cm^{-1}) and Amide II (1525 cm^{-1}) stretching which are both common signatures of the protein. [1, 2] The additional broad peak at 1100 cm^{-1} is attributed to unremoved TX-100. [1] Characteristic peaks of polypyrrole formation were observed in the FTIR spectrum of the polymer, most notably are the peaks of sp^2 C-H stretches (957 cm^{-1}), aromatic ring bending (1150 cm^{-1}), and C-N stretches (1280 cm^{-1}). [3, 4] The features from these two individual spectra are used to compare similarities observed in the spectrum of the PSI-PPy composite. Amide I (1630 cm^{-1}) and Amide II (1525 cm^{-1}) stretches help to confirm the presence of PSI, while the broader peaks of C-N stretching (957 cm^{-1}) and sp^2 C-H stretching (957 cm^{-1}) show the presence of Ppy. The most striking difference in the composite spectrum is the sharp signal at 1150 cm^{-1} that aligns with stretching attributable to aromatic ring bending of the pyrrole moiety. While this is somewhat different from the broader, less intense peak in the chemically polymerized Ppy, our hypothesis assumes that the photopolymerization provides a sufficiently unique polymerization scheme to allow the resulting polymer to accommodate more of these specific bendings in comparison to the rest of the potential stretches through the chain. Through the comparison of pure PSI and chemically polymerized Ppy spectra with the proposed PSI-PPy composite spectrum, many similarities were drawn to establish confidence that PSI did polymerize PPy.

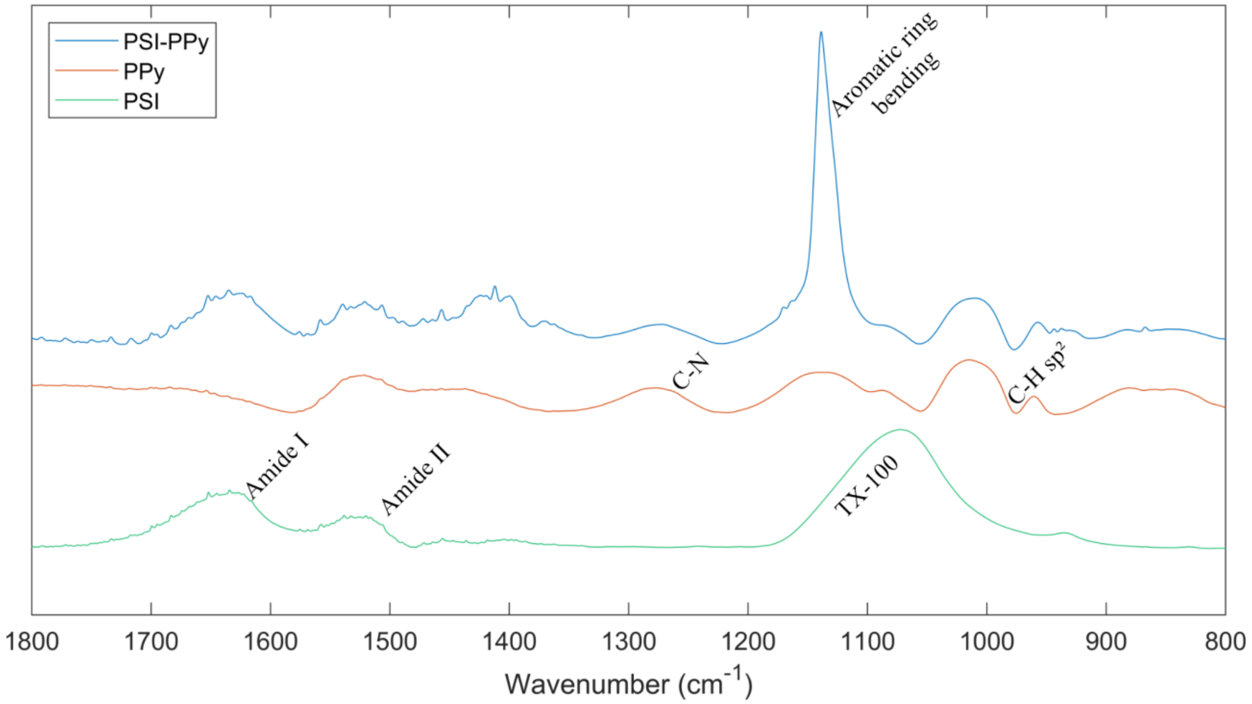

SI1: FTIR-ATR spectra of PSI (bottom), chemically polymerized PPy (middle), and PSI-PPy (top). Amide I and Amide II stretches observed in both PSI and PSI-PPy are utilized to confirm the presence of PSI whereas C-N and sp^2 C-H stretches (seen in both PPy and PSI-PPy spectra) support the growth of polypyrrole.

Additional time points of microscopy characterizations of PSI/PPy nanocomposites are shown below in **SI2.** Here the morphological evolution from barnacles to spheres is expanded, supporting the images found in the main text (**Figure 5**). The sphere sizes observed at 10 hours of illumination are showed in the histogram of **SI3**. Particles averaged around 1 μm in diameter

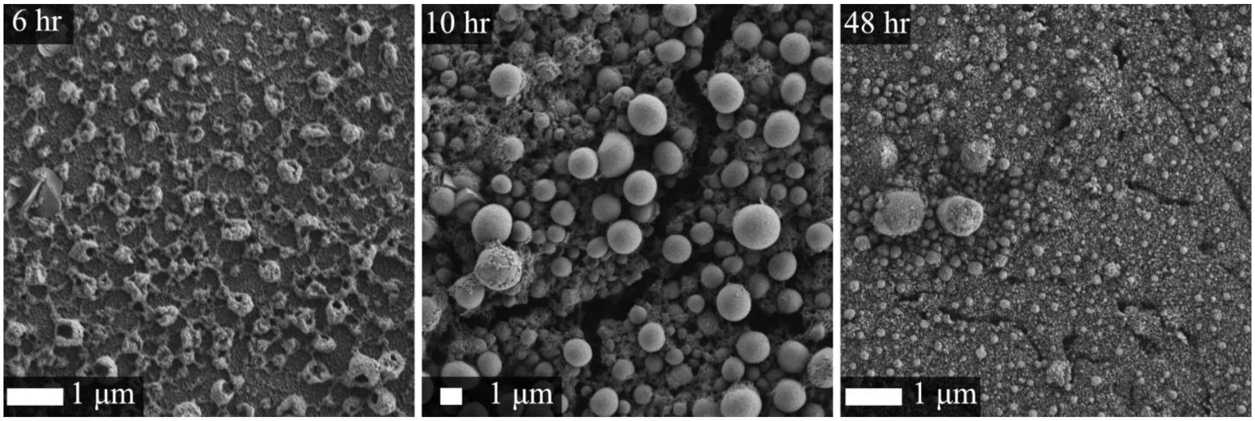

SI2: Additional SEM images of PPy/PSI films showcasing morphology changes over time. At 6 hours, barnacle density has increased from 4 hours. By 10 hours, spheres are present and by 48 hours, they have completely taken over the polymer landscape.

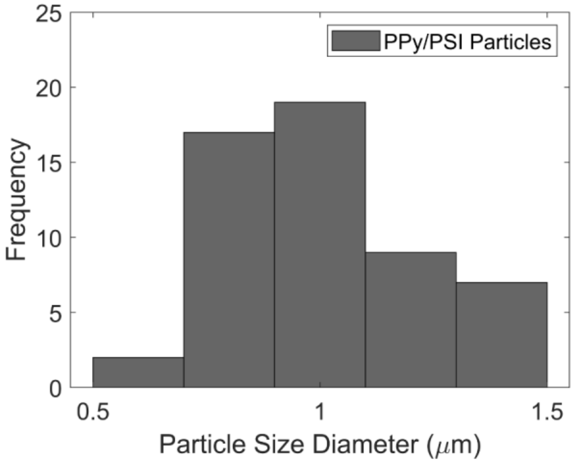

SI3: Histogram plot of PPy/PSI particle diameters from polymerization illumination times of 10 hours.

A full EDX spectrum of a PSI-PPy particle is shown below in **SI4**. The presence of the sulfur signal is used to track PSI throughout the particle. Other elements and their origins are addressed within the caption. A comparison with **SI8** helps to confirm that sulfur is not simply background noise.

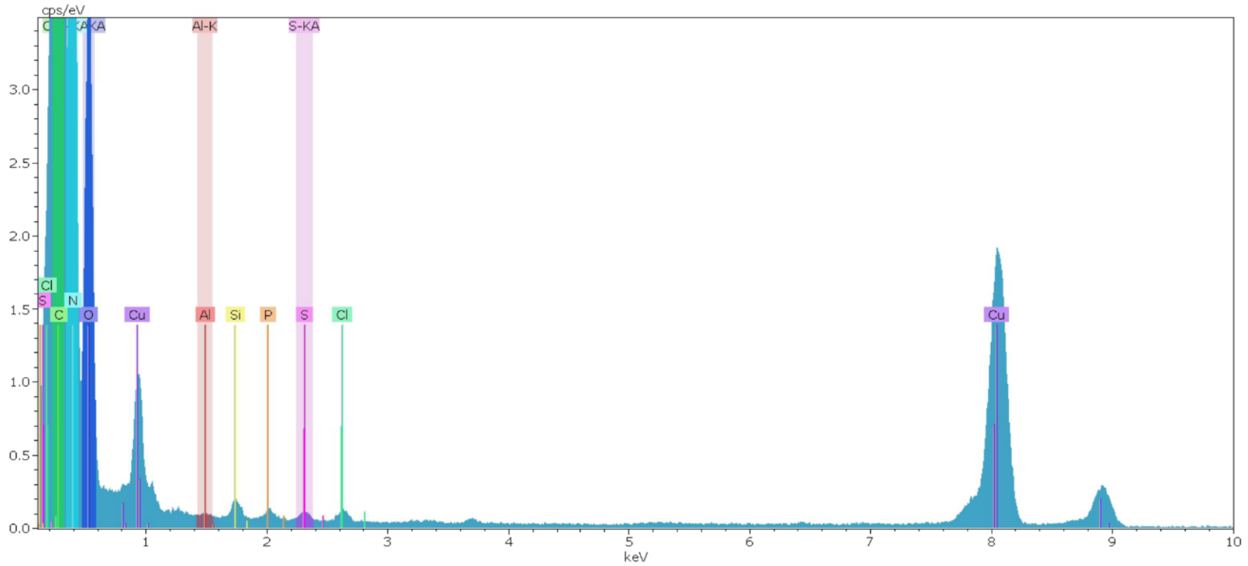

SI4: STEM EDX spectrum of PPy/PSI nanoparticle corresponding to **Figure 6** showcasing **s**ulfur content. Other elements and their source: copper (TEM grid), phosphorus (PSI buffer), chlorine (PPy counter anion), silicon (glass substrate).

TEM images in **SI5** showcase other isolated features from the PSI-PPy film. The image on the left is reminiscent of the barnacles with its hollower center whereas the thin, porous material in the center micrograph could be an isolated sheet from the more honeycomb-like structure. And finally, the particle in the right pane is simply a more traditional particle observed in the landscape on the PSI-PPy film. **SI6** provides the corresponding STEM/EDX images providing further evidence that PSI (tracked by elemental sulfur) was incorporated throughout all levels of the film.

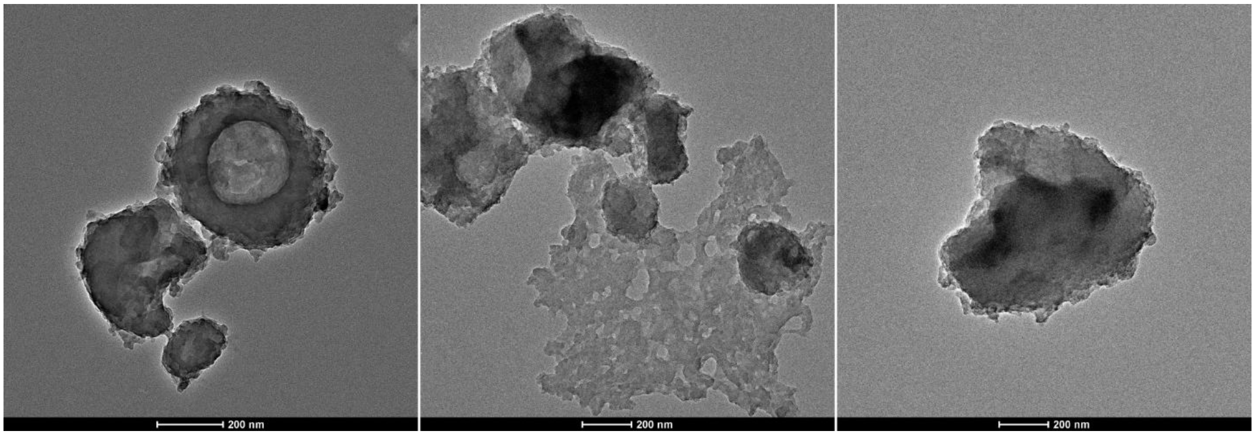

SI5: TEM images of other PPy/PSI film morphologies. From left to right: Baskets which are remnants of the column like structures, webbing from the initial honeycomb layer, and less spherical nanoparticle.

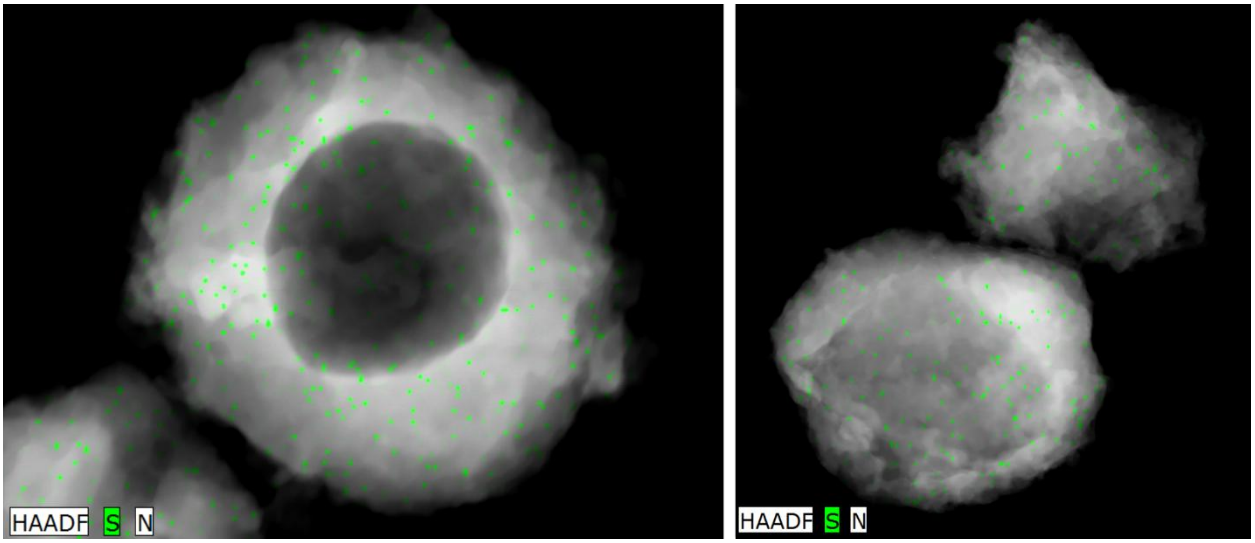

SI6: STEM EDX of a PPy/PSI nanobasket(left) and a set of generic nanoparticles (right) with sulfur highlighted in green as a marker for PSI presence.

Polypyrrole nanoparticles were synthesized without the use of PSI for comparison to PSI-assited to PPy. The methodology for synthesis is provided in the main text. Imaging and characterization of the nanoparticle products are provided below. In **SI7,** the TEM image on the left shows the monodispersity of the sample whereas the middle and rightmost micrographs showcase the finer details of a single particle. The dark spots in the TEM image (and bright spots in STEM) correspond to the higher absorption element iron which was used in the original synthesis. The presence of iron can also be confirmed by the EDX spectrum in **SI8.** More interestingly though is the lack of sulfur signal which helps to confirm that the signal seen in **SI4** must be originating from PSI.

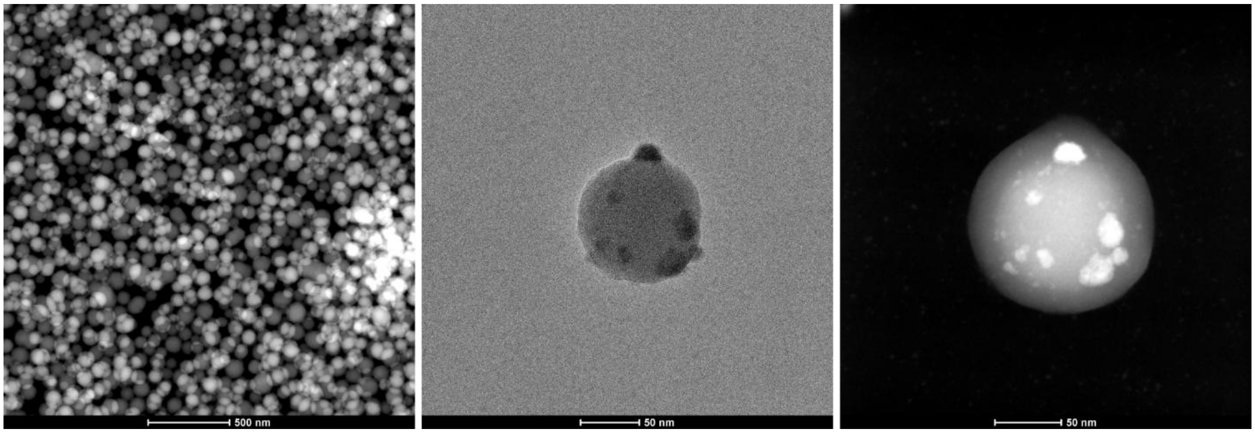

SI7: TEM of PPy/PVP nanoparticle. Left: Image of undiluted reaction mixture showcasing monodispersity of the sample. Middel and right: TEM and STEM of a single PPy/PVP particle respectively. The dark and light spots are pockets of iron from where the PPy nucleated.

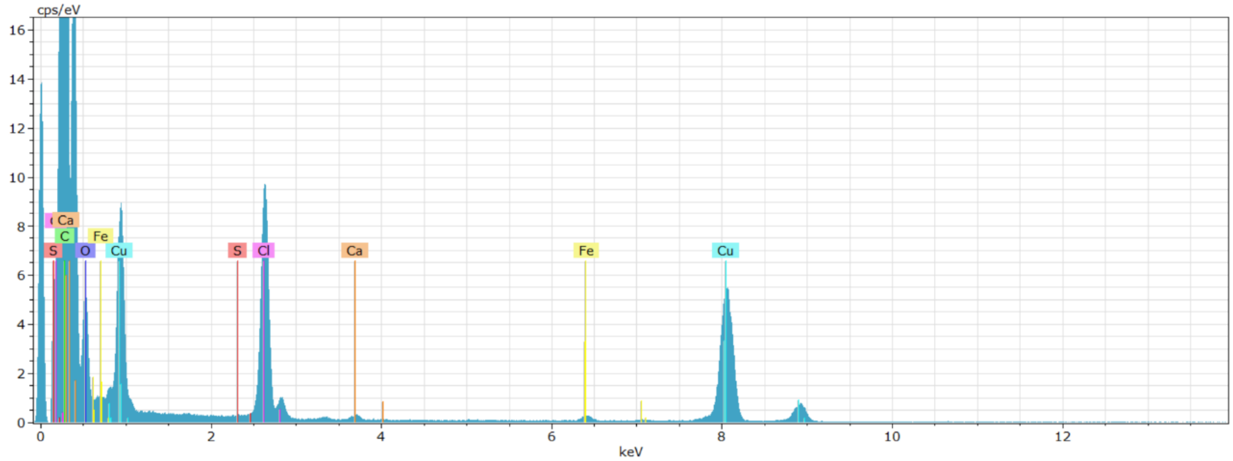

SI8: EDX spectrum of single PPy/PVP particle in **SI5**. Notably, there is not sulfur signal present. Elements of interest and their source: copper (TEM grid), iron (oxidizing agent in synthesis), chlorine (counter anion for PPy).

References

(1) Nabhan, M. A.; Cordova-Huaman, A. V.; Cliffel, D. E.; Jennings, G. K. Interfacing poly(*p*-anisidine) with photosystem I for the fabrication of photoactive composite films. *Nanoscale Adv.* **2024**, *6* (2), 620-629.
(2) Passantino, J. M.; Williams, A. M.; Nabhan, M. A.; Cliffel, D. E.; Jennings, G. K. Photooxidative Polymerization of Pyrrole from Photosystem I Proteins. *ACS Appl. Polym. Mater.* **2022**, *4* (10), 7852-7858.
(3) Tabačiarová, J.; Mičušík, M.; Fedorko, P.; Omastová, M. Study of polypyrrole aging by XPS, FTIR and conductivity measurements. *Polym. Degrad. Stab.* **2015**, *120*, 392-401.
(4) Turczyn, R.; Krukiewicz, K.; Katunin, A.; Sroka, J.; Sul, P. Fabrication and application of electrically conducting composites for electromagnetic interference shielding of remotely piloted aircraft systems. *Compos. Struct.* **2020**, *232*, 111498.
